# Publically Funded Recreation Facilities: Obesogenic Environments for Children and Families?

**DOI:** 10.3390/ijerph7052208

**Published:** 2010-05-04

**Authors:** Patti-Jean Naylor, Laura Bridgewater, Megan Purcell, Aleck Ostry, Suzanne Vander Wekken

**Affiliations:** 1 School of Exercise Science, Physical and Health Education, University of Victoria, PO BOX 3015, STN CSC, Victoria, BC V8W 3P1, Canada; E-Mails: LBridgewater@ottawaheart.ca (L.B.); mpurcell@ualberta.ca (M.P.); suzannev@uvic.ca (S.V.W.); 2 Department of Geography, University of Victoria, SSM, B203, 3800 Finnerty Road (Ring Road) Victoria, BC V8P 5C2, Canada; E-Mail: ostry@uvic.ca

**Keywords:** public recreation facilities, food environment, healthy eating, nutrition policy

## Abstract

Increasing healthy food options in public venues, including recreational facilities, is a health priority. The purpose of this study was to describe the public recreation food environment in British Columbia, Canada using a sequential explanatory mixed methods design. Facility audits assessed policy, programs, vending, concessions, fundraising, staff meetings and events. Focus groups addressed context and issues related to action. Eighty-eighty percent of facilities had no policy governing food sold or provided for children/youth programs. Sixty-eight percent of vending snacks were chocolate bars and chips while 57% of beverages were sugar sweetened. User group fundraisers held at the recreation facilities also sold ‘unhealthy’ foods. Forty-two percent of recreation facilities reported providing user-pay programs that educated the public about healthy eating. Contracts, economics, lack of resources and knowledge and motivation of staff and patrons were barriers to change. Recreation food environments were obesogenic but stakeholders were interested in change. Technical support, resources and education are needed.

## 1. Introduction

Alarming and relatively recent increases in childhood obesity [1–3] have fueled public health action in British Columbia (BC), Canada and globally [3]. It is well accepted that the current environment predisposes both children and adults to sedentary lifestyles and over consumption of energy dense foods of low nutritious value [3–5]. An obesogenic environment has been defined as a place where the influence of the surroundings and social context combine to promote obesity in populations or individuals [6]. Unfortunately, the potential health and social consequences of the development of obesogenic environments are far reaching [3,7].

There is growing recognition in the public health sector that solutions to childhood obesity should be more ecological in nature; recognizing the interplay between individuals and multiple environments and levels of influence from friends and family, to community and provincial policy [3,8–10]. Recent obesity prevention recommendations identify public service venues, including recreation facilities, as environments in which to increase access to healthy food options [11].

In British Columbia (BC), Canada, for instance, approximately fifty million visits to recreation facilities occur annually [12]. This is the equivalent of thirteen visits per British Columbian per year. Many of the benefits of recreation facilities cited by users and non-users alike are the activities for children, youth and families and evidence shows that children are spending more time in structured activities (e.g., sports and arts) or daycare than in previous years [13]. As such, recreation facilities have become a potential environment/setting of influence on children’s health behaviour [13]. Anecdotal reports and observations have suggested that there are many exposures to energy dense, low nutrient foods in the recreation environment, but there is limited evidence in the literature to support these claims. One Canadian study completed in 2006 looked at the foods offered in sports, recreational and cultural facilities in Quebec City and found that most items in the vending machines and snacks were of low nutritional value [14]. Some evidence suggests that public recreation staff are willing to collaborate with health promotion professionals to advance shared goals for physical activity promotion [15], which could also be extended to address nutritional concerns. The purpose of this study was to describe the public recreation food environment and explore the current context and facilitators and barriers to change.

## 2. Methods

We used a sequential explanatory mixed methods research design [16] with quantitative data from a facilities food environment audit tool collected first and qualitative focus group data collected second. We integrated data during the interpretation phase.

### Municipal Recreation Facilities Food Environment Audit Tool (MRFEAT)

Recreation staff completed a written audit tool called Municipal Recreation Facilities Food Environment Audit Tool (MRFEAT) to assess policy, programs and practices and the food and vending services in their facility. MRFEAT was developed based on a survey about food sales in schools [17] Heart Check^TM^ [18] and Checklist of Health Promotion Environments in the Workplace (CHEW) [19] and developed in collaboration with staff in a local recreation center and key recreation and health stakeholders. The audit tool was then piloted in seven local recreation centers and revised prior to implementation. The audit tool was 15 pages long, and divided into seven main sections. Table 1 provides a sampling of the questions for each of the audit tool sections.

### Focus Groups

Focus groups explored the current context and the needs of recreation service providers in order to determine facilitators and barriers to changing the food environment. Themes were examined in relation to implementation theory and a micro-implementation approach was used, as the focus was on how changes could be applied within the recreation environment [20]. Ethical approval was received from the University of Victoria Ethics Review Board.

### 2.1. Sample

We recruited a census sample of recreation facilities registered with the British Columbia Recreation and Parks Association (n = 216 after duplicate addresses and ineligible facilities were removed). There were 77 completed audits returned (36%). In some cases these audits represented a group of facilities (101 facilities). We used purposive sampling to identify recreation centers to participate in the focus groups. Based on advice from the MRFEAT provincial advisory committee, we selected facilities that provided maximum variation in context: geographical location, socio-economic status (SES), size and type of facility [21]. Seven focus groups were conducted: four in the Lower Mainland, one on Vancouver Island, and two in the Northern Health Authority of BC. Recreation facility managers at each center recruited staff to participate in each focus group, which ranged from eight to fifteen people, with n = 56 participants in total. Participants included recreation staff, managers, city council members, food service staff, health authority members, sport team managers, and concession operators; all of whom resided in BC, Canada. Participants agreed to take part in the study through informed consent. Two researchers from the University of Victoria traveled to conduct the focus groups, which were approximately one and a half hours in duration; participants were provided with refreshments.

### 2.2. Procedures

The MRFEAT audit tool was developed in May 2005, and was pilot tested in December 2005. The audit tool was designed to identify the range of food services and policies in place in recreation facilities across the province. It was drafted in consultation with recreation staff, and was refined. It was then pilot tested in seven municipal recreation facilities (rinks, pools, multi-use facilities) in Victoria, BC. The tool was modified based on this pilot and then sent out to the municipal recreation facilities. The provincial survey using the audit tool was conducted between March and October 2006. Dillman’s Total Survey Design Method was used to enhance response rates [22]. Two notices were sent out via the BC Recreation and Parks Association Communiqué prior to the mail-out. One month following the mail-out, a postcard was sent out to non-respondents and then two follow-up phone calls were made over the next four months.

Focus group data were collected in February and March of 2007. The facilitators were trained in conducting focus groups. The focus group questions were pre-tested for comprehension and wording [23] with the MRFEAT provincial advisory committee. Semi-structured focus group interviews were held at the recreation centers. All focus group sessions were audio taped with the permission of participants.

### 2.3. Data Analysis

The MRFEAT quantitative data was entered into SPSS for analysis and data from open-ended questions was entered into Excel for hand counting. Audiotapes from focus groups were transcribed verbatim. NVivo 2.0 qualitative software was for data analysis. Member checks were conducted by giving focus group participants the opportunity to review their transcript for verification and contact the researcher at their convenience if they had any additional comments. Line by line coding was completed for all transcripts, from which themes were then generated. Quotes and descriptions are included in the findings to provide examples of where conclusions were drawn from.

## 3. Results and Discussion MRFEAT Audit Tool

**Figure 1.**
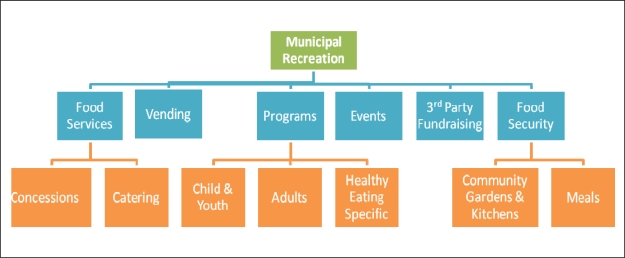
Operational areas within municipal recreation facilities where opportunities for food and beverage policy, provision and promotion are present.

The results from the audit tool identified the current state of food services, policies and programs in BC recreation facilities. Figure 1 shows a summary of the operational areas within municipal recreation facilities where food and beverage policy, provision and promotion are present and where opportunities for change towards healthier choices are possible.

### 3.1. Healthy Eating Planning and Policies

Very few recreation facilities had a healthy eating committee in place to manage corporate relations, programs or food services (8%); however, according to the focus groups some facilities were beginning to form committees, or include a nutrition sub-committee in staff planning and advisory committees. Most facilities (88%) did not have policies or standards in place to govern the types of food sold or provided for child/youth programs on site. If policies existed, they typically addressed allergy issues, although a limited number of facilities (2) indicated that they were in the process of developing policies or standards related to healthy choices.

### 3.2. Corporate Relations (Contracts, Incentives, Advertising, Control)

A large proportion of recreation facilities had food and beverage contracts (76%) and of these 70% also reported having control over food sold in the facility. In addition, many facilities had food and beverage advertising on site (47%), which is often a component of service contracts. Financial and corporate incentives (17%) and sponsorship (26%) affected a smaller proportion of facilities.

### 3.3. Snack Bars/Cafeteria

Many recreation facilities had a cafeteria or snack bar on site (61%), and these were largely open during the peak times for child visits to the facilities; 56% of these food outlets were open after school, 62% in the evening and 89% on the weekends. Snack bar and cafeteria services were operated in a variety of ways: most commonly by private companies (48%), municipality staff (36%), or sports teams (9%). The most commonly offered lunch item was a hot dog (19%). The most common side order was deep fried food (e.g., French fries, onion rings) (38%). The most common beverages were fruit juices or drinks (21%) and the most common desserts were cakes, pies, squares and cookies (55%) followed by muffins (15%). To compare, water represented 7% of the beverage options, salads represented 4% of all lunch entrées and 20% of side orders. Vegetables and fruit represented 10% of all side orders and yoghurt 2% of all desserts. The average price for a healthy option tended to be higher in snack bars than in vending machines.

### 3.4. Child and Youth Programs

Although 32% of facilities provided meals during childcare, most snacks were provided by staff members that purchase and serve the food or parents that provide a snack on a rotating basis. Of those that indicated that their facilities provided food during programming, 88% said they had input into meals. The key limitations to providing healthy options were: budget, preservation, storage and preparation. In addition, other limitations included concerns about: allergies, a perception that healthy options were boring or unattractive to children and a perception that other program providers have the responsibility to provide food and beverages (e.g., families or social services agencies).

### 3.5. Vending Machines

**Figure 2.**
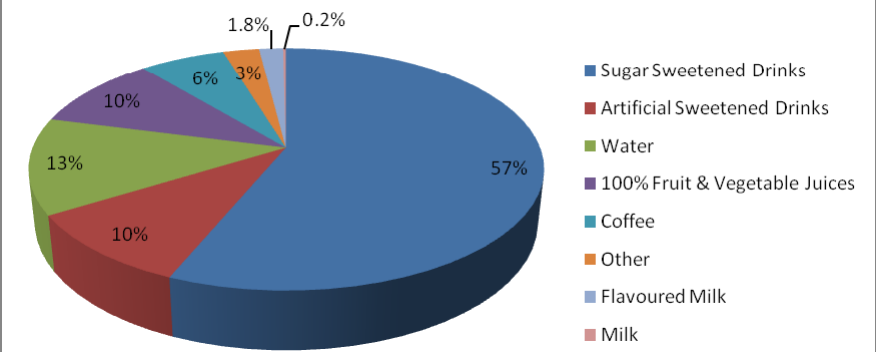
Type and proportion of vending machine beverage items sold.

**Figure 3.**
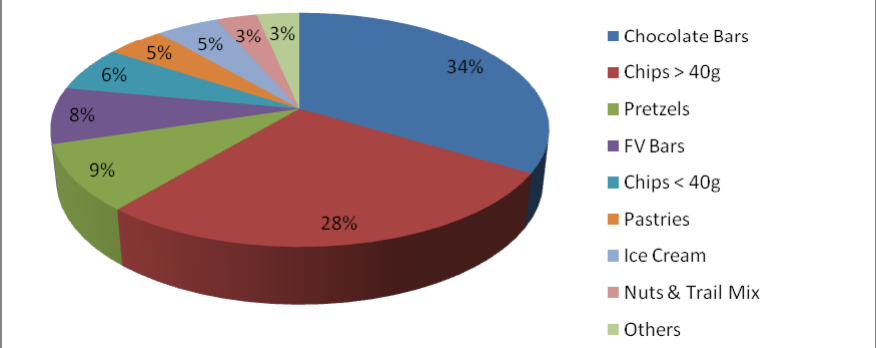
Type and proportion of vending machine snack items sold.

Vending machines were a common source of revenue for many recreation facilities. The average number of beverage vending machines per facility was 3.6 (range 0–19), while the average number of snack vending machines per facility was 1.9 (range 0–12). In 78% of facilities, children had unlimited access to beverage vending machines. On average these machines contained a majority of unhealthy options such as sugar sweetened drinks (57%) and much smaller proportion of healthy options such as water (13%), 100% fruit or vegetable juices (10%) and milk (2% flavored and 0.2% plain) (See Figure 2). In 81% of facilities, children had unlimited access to snack vending machines in which more than half the items for sale were chocolate bars (34%) and chips (34%) (See Figure 3). Although vending machines charged less for healthier options than snack bars, this was because the portion size of these products was smaller.

### 3.6. Healthy Eating Programs and Initiatives

A variety of initiatives had been implemented to promote healthy eating and decrease the sale of unhealthy food choices. The initiatives include: changes to vending and concessions, cooperation with the municipality, changes to programs, and providing public education, and integrating healthy eating planning into pre-existing healthy living or physical activity initiatives (e.g., Active Communities, a publically-funded initiative aimed at raising physical activity levels in British Columbia that mobilized and collaborated with communities, local governments, Aboriginal and partner organizations to promote healthy lifestyles choices, increase accessibility to physical activities and build supportive community environments [24]). In fact, 42% of facilities that responded (38/77) *did have* initiatives underway to educate about healthy eating such as health and wellness course or nutritious cooking classes.

### 3.7. Food Availability for Recreation Employees

Municipal recreation employees were asked about food options that were available during staff meetings (internal, external) and social events, as well as whether any food policies or guidelines exist to encourage healthier choices. Ninety-seven percent of facilities that responded (n = 65/77) did not have a food policy or guideline in place for internal meetings, 98% did not have one for external meetings, and 74% did not have guidelines for social events. When asked to rate the nutritional value of food provided at meeting and social events, most did not respond (45/77). However, of those who did respond, 43% said food served at meetings was mostly healthy. In addition to providing food at meetings and social events, 75% of recreation facilities reported that staff brought treats (*i.e.*, sweets and baked goods) from home to share with staff, which also affected the food environment.

### 3.8. Fundraising

According to the audit, recreation facilities had an average of 2.5 (sd 12.4) fundraisers per year. The most common items sold at fundraisers were hotdogs (15% of products), baked goods (17%), chips (10%), pop (9%), fruit and vegetables (8%), chocolate bars and candy (6%) and water (3%). Fundraisers held at the recreation facilities were selling non-nutritious, calorically dense food options.

## Focus Groups

The data collected from the audit broadly indicated that the food environments were not particularly healthy in most recreation facilities, but staff were interested and engaged in some improvement initiatives. In response, focus groups were conducted to gain a better understanding of the barriers and facilitators that recreation facilities may face. The information collected from the focus groups has been grouped into ten key themes, which are explained below. Both results and recommendations from the findings are presented in this section.

### Theme 1: Education

The recreation staff expressed a need to educate staff and the public on healthy eating, and making healthy choices. They felt that staff did not have adequate nutritional education necessary for selecting the most appropriate healthy choices for vending machines and concession operation. For example one staff member stated, “we need some education for food service staff”. Staff indicated that education needed to go beyond staff at recreation facilities and extend to educating the public: “we need more info to give parents and kids about healthy choices”.

### Theme 2: Food Security

Food security emerged as an important concern for many communities. The definition for food security adopted by the World Health Organization is “when all people, at all times, have physical and economic access to sufficient, safe and nutritious food to meet their dietary needs and food preferences for an active and healthy life” [25]. Food security provides conditions that promote health by providing essential nutrients and minimizing food related stress [26]. Many comments arising in the focus groups reflected concerns related to proper nutrition and basic food security issues. Although the topic arose in all focus groups, comments regarding food security were more prevalent in recreation centers situated in lower income neighborhoods. One focus group participant said, “any child, if they are hungry on the playground, we automatically feed them”. Another staff member stated that “kids can come in to the recreation centre and get food on school breaks if they have [no food]”. Since recreation facilities are places where youth frequent during their school breaks and children spend significant time before and after school, they also become regular food access locations, especially in neighborhoods where food security is an issue. With this in mind, recreation centers may benefit from access to support (information, grants and partnerships) for providing nutritious foods in their facilities, and increasing food security.

### Theme 3: Programs

Similar to the MRFEAT audit results, focus groups highlighted that some recreation centers had implemented programs that promoted and provided healthy choices in their facilities. One staff member commented on a healthy eating program they had started:

“We have child/preschool programs going on in which there are cooking classes which have switched to incorporate all the food groups and decrease the amount of cookies and treats being made, for example they cooked a vegetable curry and it was a success”.

Participants indicated that they would like to learn about best practice models and success stories from other recreation centers, in order to engage with programs that have been successful. In particular, one person requested “examples of models that are working, how, why, where and when”. Another person stated that “we need some examples of people who have done this successfully, and how they went about it”.

### Theme 4: Partnerships

Recreation centers indicated that partnerships were instrumental to the successful promotion of healthy eating. In particular, one person indicated the “need to cultivate corporate relationships that work together”. The literature indicates that partnerships are an effective way to help promote and sustain health promotion initiatives [27].

### Theme 5: Vending

Vending machines were repeatedly discussed in the focus group sessions, specifically concerning the food sold in the vending machines and the contracts with vending companies. The types of concerns raised included,

“…how is it possible to change the food in the vending machines? What is the duration that milk is going to last? How do you do it? People are concerned about if it is even possible … so they are just turning off the idea”.

Many facilities are committed to long term contracts with vending companies, therefore, resources to assist recreation centers with developing healthy request for proposals (RFPs), as well as for approaching negotiations with vending companies to offer healthier options were desired. At the time of the focus groups, the number of vending companies that were offering healthy options was limited but increasing. Some comments also focused on the importance of vending as a source of revenue for recreation centers, and the difficulty of accessing alternative funds if vending machines changed or were removed. For example, one person emphasized the need to be “point[ed]…in the direction where we won’t lose money, but maintain healthy choices”. For many recreation facilities, however, “the problem lies within the costs, unless items are subsidized [people won’t pay more for them and] the company will pull the machines unless they make some profit”. As a result, many recreation facilities felt pressured to keep sales high by selling unhealthy choices.

### Theme 6: Policy Implementation

Policy related to food and beverages was discussed as a key ingredient for ensuring healthier options were available in vending machines, at concessions, fundraising events, and in child and youth programs. One participant commented “…we really don’t have any policies in terms of what we’re going to serve to the children … nothing that is written, no procedures”. Similarly, another participant stated that they “don’t have any policies or guidelines”. From the responses, it was evident that most recreation centers did not have healthy eating policies in place; however, staff indicated that having a policy may help them to implement healthy choices. This supported the results of the MRFEAT audit.

### Theme 7: Facilitation of Change

When discussing the motivation to change in the focus groups, recreation staff indicated that change would be most successful if it was done in a slow, step by step manner. “None of this happens overnight, and we have to be patient when we are trying to create change”. Also, it was expressed that they “…should take baby steps to get the best reaction”. These comments are consistent with the body of knowledge from implementation research, that change is best accepted when done slowly [28]. Change also occurs best when stakeholders are involved in the changes that will be made [29]. Therefore when facilitating change in the recreation centers it is important to incorporate staff ideas, and involve them in the process.

### Theme 8: Environment

Focus group participants stated that the recreation environment impacts healthy eating in two ways. First, if vending machines provided unhealthy options, children would choose those options over healthier ones. For example one participant stated, “…for me it’s all about accessibility, it’s my own kids, little kids, they see a vending machine and they want something, if they don’t see it, they don’t ask for it”. Second, participants felt that providing healthier choices was imperative and that changes would have to be made in order to achieve this. The following comment reflects this: “concession stands are selling unhealthy snacks and need to be monitored”. Recreation centers needed information on how to provide healthier options in their concession stands, as well as how to improve vending options.

### Theme 9: Barriers

Many barriers and issues were raised by focus group participants as to why it was a challenge to provide healthy food options in recreation centers. Some of the typical responses included, “we are unsure of how we will plan and promote change”, and “[healthy food] takes longer to prepare and more [cooking equipment] is needed”, and finally, “accessibility of [healthy food supplies] … is another obstacle”.

### Theme 10: Facilitators

Participants had many suggestions of facilitators for offering healthier food and beverages in recreation facilities. They commented on resources that would be useful, including posters to advertise healthier choices, online resources, information on ways to provide healthier food, as well as general educational material on nutrition. One participant commented it would be useful to “maybe even start with a booklet of alternate ways to provide things, modifications to things you might be selling that are healthier”.

## 4. Conclusions and Recommendations

Publicly funded recreation is a critical setting for action on healthy eating. To our knowledge this is the second published study about this important environment and its potential to support public health goals. Our data highlighted however, that recreation environments were not offering or promoting healthy choices and in many cases promoted food and beverages that are considered major culprits in the increasing obesity epidemic. There was a strong interest and motivation among select recreation facility staff and stakeholders to change their situation. The recreation facilities expressed that changing the food environment would require support, and would not happen quickly. Implementation would need to be founded on inclusion of recreation stakeholders throughout the process and support for change. In addition, a resource toolkit and other supports for action were necessary to address barriers and facilitators to change and successfully change the food environment in recreation.

Since the majority of facilities had food and beverage services on site and reported having control over what food items were sold, opportunities existed for staff to influence the nutritional quality of food services. However, few facilities had committees or initiatives underway to help promote and provide healthier choices and the focus group results challenged the notion of control over the food items sold when long-term vending contracts were in place and there was potential for loss of revenue. In addition, children’s nearly unlimited access to snack bars and vending machines suggests that changing products as well as limiting access to unhealthy food choices may be recommended. Although few facilities offered meals to children in programs, staff had many opportunities to influence the snacks served, as well as educate parents about sending healthy lunches. The implementation of food policies in recreation would support the efforts of staff in initiating healthy changes to the food environment, and should be pursued further.

The results from the focus groups provided in-depth information and feedback related to staff perceptions about promotion and provision of healthy food in the facilities. From these discussions it is evident that recreation staff required increased education and support for healthy eating, and that it was important that they understood the rationale for the changes. Furthermore, many communities faced food security issues. Some facilities offered programs that promoted or provided healthy food; however, the majority did not, so this may present another opportunity for recreation to support community health.

Information on healthy eating and food security initiatives or programs that are currently being implemented in recreation centers should be shared with other recreation centers. This transfer of knowledge may motivate and assist other recreation centers in adopting programs that have been successfully implemented elsewhere.

The strengths of this study were the mixed methods design that allowed for corroboration across data sources and participants as well as in-depth discussion with stakeholders. These findings are limited by the self-report nature of the data, high variability across facilities and a low response rate that may have led to a positive response bias and consequently an overstatement of the situation. The response rate was attributed to the length and difficulty of the audit and the timing during which the audit was completed (following 2 years of infrastructure surveys).

Data on changing the food environment in recreation facilities is limited, and the one recent study of sports, recreational and cultural facilities in Quebec City [14] confirmed the findings of this study—a majority of foods and beverages served had low nutritional value and there were a limited number of healthy choices.

This study validates the need for further research on effective setting-based approaches to changing food environments in recreation facilities. The impact of environmental change interventions on food products, sales and patron responses within the context of municipal recreation should be researched further. The data gathered in this study indicates the need for policy, adequate resources, partnerships and information to design and facilitate healthier choices in municipal recreation facilities.

## Figures and Tables

**Table 1.** Sample questions from the BC municipal recreation facilities food environment audit tool.

**Survey Section**	**Sample Questions**
A. Food Provision	Does your facility have a food or beverage service or other food related contracts with any suppliers? What type of food selling facilities are on site? Are children in out-of-school or daycare programs provided meals or snacks on site?
B. Vending Machines	How many beverage/snack vending machines are in the facility? For each machine, count the number of selections in each category provided and record this number in the table.
C. Cafeteria Snack Bar	How often are items with whole grain bread, buns, or pizza crusts offered? Please indicate always, sometimes, rarely or never. Please conduct *a “walk through”* of the cafeteria/snack bar at lunchtime and complete the following table. List the food and beverage items served by the categories: lunch entrées, side orders, beverages, dessert/bakery items.
D. Food Sale Fundraisers	During *2005,* how many food sale fundraisers (hot dog days, pizza days, PAC lunch) were held at the facility? Please list the main foods (hot dogs, pizza *etc*.) and the supplementary foods (pop, chips, ice cream, *etc*.) served at each of the food sale fundraisers during *2005.*
E. Facilities Food Policies and Guidelines	Is there a committee in place to promote healthy eating at the facility? Do you have any policies or standards in place governing the type of food sold or provided for child/youth programs on site? If so, please describe these and provide a copy.
F. Programs and Initiatives	Do you have any programs or initiatives (day, evening or after-school) underway to educate children or the public about healthy food choices? Please list and describe.
G. Availability of Food for Municipal Recreation Employees	Rate the nutritional value of the food provided at meetings on a scale of 1–5 (1 = none or almost none of the food meets nutritional guidelines and 5 = all food provided meet nutritional guidelines) Do you work colleagues bring sweets from home and leave it in the employee’s kitchen for everyone to eat?
